# Apathy as Marker of Frail Status

**DOI:** 10.1155/2012/436251

**Published:** 2012-02-12

**Authors:** Roberta Semprini, Adele Lubrano, Giulia Misaggi, Alessandro Martorana

**Affiliations:** ^1^Clinica Neurologica, Dipartimento di Neuroscienze, Università di Roma “Tor Vergata”, Via Montpellier, 00133 Rome, Italy; ^2^IRCCS Santa Lucia, Via Ardeatina, 00179 Rome, Italy

## Abstract

Frailty is a complex and dynamic condition associated with aging. This condition is characterised by the difficult adaptation of an old subject to new challenges occurring during life. Frailty is supposed to be due to the progressive decrease of physiological reserves and multiorgan and multisystem change. It coincides with a reduced or absent resilience. In general comorbidities like hypertension, heart disease, inflammation and infectious diseases are potential risk factors for and psychophysical decline. The aim of this work is to highlight the importance of impaired cognition as factor predisposing to frailty. The authors are convinced and suggest that the presence of neurobehavioral disturbance like apathy associated to impaired executive function could be the major predisposing factor for frailty and unsuccessful aging. Unfortunately available literature largely underestimates the presence of these factors. Thus to better identify markers of frailty, a good neuropsychological assessment and the evaluation of behavioural disturbances are suggested.

## 1. Introduction

Aging is a complex phenomenon which features have recently gained interest. In general aging is characterised by the progressive accumulation of deficits taking place in different individuals and in different pathways (molecular, cellular, psychological, and psychosocial, etc.), and with a variety of rates proper of each individual, that may depend on the interplay between intrinsic and extrinsic factors [1, 2].

 Associated to aging we recognise a nonspecific condition defined “frail-old” or “frailty syndrome”. This is a condition that has long been considered synonymous of disability and comorbidity since it is prevalent in old age. Frailty is a condition that expose an old subject to be vulnerable to many non threatening-illness conditions, although they are not recognised as precipitating factors [3, 4].

A good definition of normal aging, even in general terms [5], may be useful to better understand the concept of frailty and its features.

Normal aging is widely considered a physiological condition during which natural reserves progressively decrease, while still supporting an acceptable functioning of essential organs, in a steady state. Thus aging is considered a dynamic event in which a progressive dysregulation of homeostatic conditions makes the organs and systems less or nonresilient [6, 7].

 Many variables (biologic, metabolic, psychosocial, etc.) may play a significant role in the evolution of aging process. The association of these variables, which are different among individuals, with comorbidities (hypertension, heart disease, diabetes, bone fractures, infectious diseases) are considered responsible for the quality and rate of aging. Thus, the absence of disabilities and unimpaired cognition might be considered the features of successful aging [8]. Conversely, unsuccessful aging could be the result of the association between disabilities and cognitive decline. Considerable evidence points to impairment of executive function as a key contributor to age-related declines in a range of cognitive tasks [9].

Therefore, frailty could be considered a dynamic condition set between successful and unsuccessful aging.

Following the “Frailty Task Force” of the American Geriatric Society frailty has been considered a clinical syndrome defined by the presence of 3 or more of the following symptoms: unintentional weight loss, self-reported exhaustion, weakness, slow walking speed, and low physical activity. Clinical signs are represented by undernutrition, sarcopenia, balance and gait disorders, and osteopenia. These features are expected to hesitate in increased risk of falls, worsening mobility or reducing it.

Several conditions have been identified as possibly related to a frail status and thus responsible almost in part for decline of old person.

Aging itself is generally accepted to be accompanied by altered metabolic processes like increased free radicals production, telomere shortening, and mitochondrial dysfunction; changes associated to extrinsic conditions like inflammatory status (IL-6, TNF; CRP) and common comorbidities (chronic illness like diabetes, hypertension, coronary heart disease, obesity, malignancies) may be responsible for frailty and life-shortening [10–14]. In this view, frailty may be considered as a condition which precedes in a subclinical manner, such that when resilience is overwhelmed it becomes manifest concomitantly with the appearance of chronic illness. At the same time altered resilience could be the result of altered volition and motor planning, thus the result of impaired executive functions. This in turn could be responsible for hypomobility and reduced autonomy and as a consequence might render someone frail that successfully aged. In this view impaired cognition may be a precipitating factor that shifts a subject from the prefrail to the frail status [15].

The aim of this work is to discuss the role of altered cognitive conditions of aged person and in particular we will focus on executive functions and apathy in aging.

## 2. Executive Functions

Cognitive impairment is the primary determinant of disability in late life, and at all ages, cognitive function is the foundation of individual capacity to meet the challenges of any disabling condition [16]. 

Executive functions refer to a variety of higher cognitive processes that are related to the function of frontal lobes. In general this area of the brain is able to integrate the formation flow arising from many sensory systems set in both anterior and posterior brain, necessary for goal-directed action and also for the control of attentional resources which are considered basic for daily living activities [17]. In general anterior parts of frontal lobes are involved in aspects of self-regulation (inhibition or self-awareness) while posterior parts are involved in reasoning process [18].

PFC and striatum are the main regions of the frontal lobe involved in the control of executive functions [19, 20]. In both regions dopaminergic system plays an essential role as a modulator of cortico-striatal functions, acting through its receptors, namely, D1-like and D2-like [21–23]. Changes of the dorsolateral prefrontal-caudate circuits are considered responsible for disexecutive function, which lead mainly to a subcortical dysfunction. Frontal lobes are involved in the pathophysiology of many neurological and psychiatric disorders and are also highly susceptible to changes of aging [15, 24, 25].

During normal aging synaptic changes of frontal cortex and associated to changes of the striatum (like altered dendritic pruning, decrease of neurotransmitters efficiency, in particular of dopaminergic system, and also the presence of diffuse white matter lesions) [26] are considered responsible for poor performance on executive tasks in healthy old subjects, as demonstrated in neuropsychological studies. Moreover executive functions contribute also to motor planning and therefore to maintain gait stability. As a consequence an impairment of pre-frontal-striatal circuit could be also responsible of gait instability and slow walking speed observed in healthy old subjects.

Gait instability and slow speed during walking are considered risk factors for falls and disability.

Thus it is conceivable to suggest that, as depicted previously about the features of frail patient, impairment of executive functions during normal aging could represent the marker to distinguish a successful aging from a prefrail status, however not sufficient to determine frailty of a subject.

Our suggestion is that, more importantly, the presence of apathetic behavior could represent reasonably a precipitating factor for frailty.

## 3. Apathy and Aging

Apathy is a behavioural syndrome common in normal physiological aging and is also part of the psychiatric spectrum of mental illness, and of many neurodegenerative disorders like Alzheimer's disease Apathy is an observable behavioural syndrome consisting in a quantitative reduction of voluntary (or goal-directed) behaviours [27]. Therefore, apathy occurs when the systems that generate and control voluntary actions are altered. In this view apathy can be defined as the quantitative reduction of self-generated voluntary and purposeful behaviour and necessarily has not to be considered a clinical aspect of depression (see [28, 29]).

In general the basal ganglia and their connection with the prefrontal cortex are deputed to decision making. Hence, any dysfunction of this frontostriatal circuit may be responsible for apathetic behaviour.

Anatomical circuits of apathy are represented by cortical areas like the prefrontal cortex (neo-, paleo-, and archeo-cortex, amygdala, and hippocampus) and the ventral basal ganglia (limbic striatum or better the nucleus accumbens, midbrain ventral tegmental area, medial tip of subthalamic nucleus, centromedian, and parafascicular nuclei of the thalamus) [19, 30–32]. The roles played by prefrontal cortex (PFC) and by nucleus accumbens (NAcc) render these structures as the most involved in the appearance of apathy [33, 34].

PFC has an essential role in cognitive and executive processes that involve motivation, emotion learning, and memory. PFC integrates sensory and limbic information and promotes goal-directed behavior through efferent projections to the NAcc. In addition, PFC sends outputs to other limbic areas such as the hippocampus and amygdale, which in turn modulate the activity of the NAcc through excitatory-glutamatergic projections.

**Figure 1:**
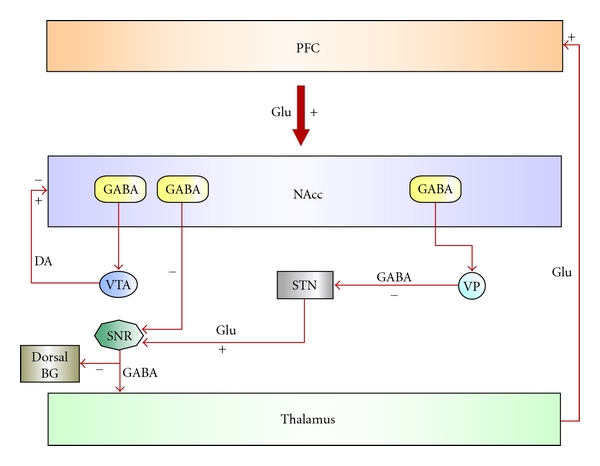
Schematic representation of basal ganglia circuit. In this scheme the nucleus accumbens (NAcc) is an interface through which limbic (glutamatergic) structures influence motor activity, in a way that limbic structures can influence behavior, under control of meso-limbic (dopaminergic structures) system (Ventral Tegmental Area neurons).

NAcc receives inputs from cortical areas (neocortex, hippocampus, amygdala) and envy inputs to the ventral pallidum, which represents the output nuclei of the ventral basal ganglia system. This is a region that through its connection with the thalamic nuclei transfers information back to the cortex. This arrangement is made to select relevant signals from background noise arising from multiple inputs and to transfer it back to the cortex in order to generate output signal to target nuclei. Put in this frame NAcc has been proposed to play a role in emotion, and more generally in limbic-motor integration (see Figure 1). This hypothesis has been based on the anatomical organization of the NAcc which suggests that this nucleus is an interface through which limbic (glutamatergic) structures influence motor activity, and that these limbic influences on behavior could in part be controlled by mesolimbic (dopaminergic structures) system. Ideally any morphologic alteration in these brain regions can potentially induce apathetic symptoms.

Following Levy and Dubois's work [27], apathy has to be considered heterogeneous disorder that may be ascribed at least to three different phenomena, each related to a specific topography in basal ganglia. The first involves the affective-emotional processing, thus the medial PFC connection with amygdala and NAcc, the second involves the cognitive processing, thus the lateral PFC and caudate nucleus connection, and the third involves the so-called “auto-activation” processing, which is observed in the severe cases of apathy. In these cases a link with pallidal dysfunction o extensive cortical lesions was supposed.

During normal aging it is conceivable to suppose that due to morphological and metabolic changes of cortical neurons as well as of subcortical nuclei, disorder of emotional-affective processing may appear. PFC and hippocampus have been demonstrated to show particular vulnerability during normal aging. Subtle regional changes of dendritic branching or altered mechanisms of neural plasticity have been experimentally demonstrated in lab animals and also in humans [35–39]. These changes are also associated with reduced levels of neurotransmitters like acetylcholine, glutamate, GABA, and dopamine decrease with age (Chen et al. [40]). Such alterations may reasonably be responsible for appearance of apathetic behavior in old subject. In this view, several reports showed that dopamine transmission is particularly vulnerable with age, in particular reduction of the accumbal dopamine transporter and of cortical dopamine receptors (both D1-like and D2-like, where D2-like seems to be prevalent) in aged subjects ([41], Volkow et al. [42]; Ishibashi et al. [43]; and Bäckman et al. [44]). These changes were related to PFC cognitive deficits and in particular were related to executive function impairment [45]. Even reward processing which has been demonstrated to be tuned by dopamine is altered in aged subjects [44, 46, 47]. Thus, dopamine appears particularly as vulnerable to aging. Given the particular deficits of dopamine transmission and the role played by this transmitter in the control of PFC-basal ganglia circuit, it is conceivable to suppose that such changes could be responsible for apathetic behavior in old subject. Moreover, apathy increases with age in healthy old population [48], and its presence is considered an early sign of cognitive decline [49].

## 4. The Apathy as Marker of Frail Status

Prefrail or frailty syndrome is considered dynamic conditions where the reduced resilience of a subject might induce changes responsible for disability, hypomobility, and even death.

Certainly the absence of disabilities and also preserved cognition, as mentioned previously, represent the target for a good aging.

Population-based studies performed in old healthy adults demonstrated that only 30% of population examined can be defined as successfully aged [8]. About 70% of subjects unfortunately suffer from several disabilities that may shorten their life.

Certainly disabilities like diabetes, hypertension, and heart disease of infections are easily identified and often patient's history shows how these were able to interfere with aging compromising the possible recovery. Sarcopenia, undernutrition, and inflammatory status are conditions that may be easily measured [3, 4].

Conversely cognition and neuropsychiatric disturbances in healthy old subjects are infrequently evaluated among old healthy subjects [50–52]. They are considered typical disturbances of patients with frank cognitive decline and thus evaluated among cognitively impaired patients, and rarely among normally aged subjects [53]. Such condition renders the weight of impaired cognition and of apathy on nondemented healthy subjects underestimated.

Recently few studies [25, 54, 55] outlined the necessity for measures of cognitive abilities and of neuropsychiatric inventory among aged subject. Indeed, the presence of apathy during aging might be responsible for hypomobility and reduced volition on one hand; on the other hand it may be the principal cause of carelessness and as a consequence of progressive worsening of comorbidities.

In this view, the presence of apathy, which we are strongly convinced that it is underestimated among aged adults, could represent the marker of a prefrail status predisposing a subject to become frail.

From our consideration emerges the need for further studies to better identify the real impact of apathy and impaired executive functions on frailty syndrome.
